# Evaluation of the performance of GPT-3.5 and GPT-4 on the Polish Medical Final Examination

**DOI:** 10.1038/s41598-023-46995-z

**Published:** 2023-11-22

**Authors:** Maciej Rosoł, Jakub S. Gąsior, Jonasz Łaba, Kacper Korzeniewski, Marcel Młyńczak

**Affiliations:** 1 Faculty of Mechatronics, Institute of Metrology and Biomedical Engineering, Warsaw University of Technology, Boboli 8 Street, 02-525 Warsaw, Poland; 2 Department of Pediatric Cardiology and General Pediatrics, Medical University of Warsaw, Warsaw, Poland

**Keywords:** Health care, Engineering, Mathematics and computing

## Abstract

The study aimed to evaluate the performance of two Large Language Models (LLMs): ChatGPT (based on GPT-3.5) and GPT-4 with two temperature parameter values, on the Polish Medical Final Examination (MFE). The models were tested on three editions of the MFE from: Spring 2022, Autumn 2022, and Spring 2023 in two language versions—English and Polish. The accuracies of both models were compared and the relationships between the correctness of answers with the answer’s metrics were investigated. The study demonstrated that GPT-4 outperformed GPT-3.5 in all three examinations regardless of the language used. GPT-4 achieved mean accuracies of 79.7% for both Polish and English versions, passing all MFE versions. GPT-3.5 had mean accuracies of 54.8% for Polish and 60.3% for English, passing none and 2 of 3 Polish versions for temperature parameter equal to 0 and 1 respectively while passing all English versions regardless of the temperature parameter value. GPT-4 score was mostly lower than the average score of a medical student. There was a statistically significant correlation between the correctness of the answers and the index of difficulty for both models. The overall accuracy of both models was still suboptimal and worse than the average for medical students. This emphasizes the need for further improvements in LLMs before they can be reliably deployed in medical settings. These findings suggest an increasing potential for the usage of LLMs in terms of medical education.

## Introduction

The rapid advancements in artificial intelligence (AI), machine learning (ML) and natural language processing (NLP) methods have paved the way for the development of large language models (LLMs), which possess an unprecedented ability to understand and generate human-like texts. These models have demonstrated remarkable performance in various tasks, spanning from sentiment analysis, machine translation, to text summarization and question-answering^1,2^. As a result, the potential application of LLMs in various domains, including medicine along with healthcare, is a topic of significant interest^3^. Recently, the AI topic has gained in even more general popularity thanks to the ChatGPT chatbot introduction for the public^3^.

ChatGPT is a LLM developed by OpenAI and initially released on the 30th of November 2022 on the website https://chat.openai.com/. ChatGPT became the fastest-growing application in history, as it gained 1 million users in 5 days and 100 million users just 2 months after the initial launch. The first release of the service was based on the 3.5 version of the generative pre-trained transformer (GPT) model. The model was trained using Reinforcement Learning from Human Feedback technique with Proximal Policy Optimization (PPO)^4^. The training procedure contained three steps: (1) supervised learning where the AI trainer indicated the desired response, (2) training a reward model based on the ranking of different outputs, and finally (3) optimizing the policy against the reward model using the PPO. On the 14th of March 2023, the newest version of the GPT model (GPT-4) was also released. Access to this model was restricted only to the premium users of the OpenAI chatbot (one can become a premium user only by taking out the subscription). GPT-3.5 and GPT-4 training data were cut off in September 2021, so those models were not exposed to the newest data. Both GPT-3.5 and GPT-4 performance were validated on the Massive Multitask Language Understanding (MMLU) test, which consists of multiple-choice questions from different branches of knowledge and was designed to measure knowledge encountered by the language model^5^. GPT-4 model outperformed other models not only in the English version but also after translation of the test to other languages (even those rarely used like Latvian, Welsh, or Swahili)^6^.

In order to incorporate GPT-3.5/GPT-4 into a specific field it needs to be further validated in the field-specific tests. In medicine, the expertise of healthcare professionals is crucial in ensuring accurate diagnosis, effective treatment, and patients’ safety. To maintain a high standard of medical practice, rigorous assessment methods, such as different medical final examinations, are employed to evaluate the competency of medical graduates before they begin practicing independently. Such examinations cover a wide range of medical knowledge, including theoretical concepts, clinical reasoning, and practical skills, making it a suitable benchmark for evaluating the performance of LLMs in the medical domain^7,8^. Results of validation analysis of GPT-3.5 on numerous medical examinations have been recently published^7,9–14^. GPT-3.5 and GPT-4 were already validated on, to our knowledge, several national medical tests like the United States Medical Licensing Examination (USMLE)^8^, Japanese^7^, and Chinese National Medical Licensing Examinations^12,13^, on a couple of medical benchmark databases like MedQA, PubMedQA, MedMCQA, and AMBOSS^14–16^. GPT-3.5 was also evaluated in terms of its usability in the decision-making process. Rao et al. reported that GPT-3.5 achieved over 88% accuracy by being validated using the questionnaire regarding the breast cancer screening procedure^17^. GPT-4 also outperformed GPT-3.5 in terms of soft skills tested in USMLE like empathy, ethics, and judgment^18^. Medical curricula, education systems and examinations can vary considerably from one country or region to another^19–21^. Therefore, evaluating these models with respect to European-based medical exams, including the Polish perspective, helps understand how well LLMs can adapt to specific regional requirements and standards. The examination content might vary in different regions as they might have distinct medical practices, guidelines, terminologies, and legislations, and the LLMs’ performance should align with those nuances. For example, the Polish Final Medical Examination (PFME) contains 13.5% and 7% of questions related to surgery and psychiatry, while USMLE Step 2CK contains 25–30% and 10–15% of questions from those disciplines. To the best of our knowledge, there have been no studies yet presenting the capabilities of GPT-3.5 and GPT-4 in terms of European-based medical final examinations as well as the potential of those models for such exams in the Polish language. Moreover, no other studies on the influence of the temperature parameter on medical final examination results were performed.

## Objective

In this paper, we hence aimed to investigate the utility of GPT-3.5 and GPT-4 in the context of the Polish Medical Final Examination in two language versions—Polish and English. By evaluating the LLMs’ performance on the examination and comparing it to real medical graduates’ results, we seek to better understand their potential as a tool for medical education and clinical decision support as well as an improvement of the GPT technology which comes with the newest version of the model. We also aimed to evaluate the influence of the temperature parameter on the models' responses in terms of questions from the medical field.

## Materials and methods

Polish Medical Final Examination (Lekarski Egzamin Końcowy, or LEK, in Polish), which is necessary to complete medical education under Polish law and to pass to apply for the license to practice medicine in Poland (and based on the Directive 2005/36/EC of the European Parliament also in European Union). The examination questions are prepared by the director of the Medical Examinations Center in cooperation with representatives of medical universities in Poland. Each participant can choose the language of the examination (Polish or English). The English version of the examination is translated from the original Polish one. The exam is a test comprising 200 questions with 5 options to choose from and only a single correct answer. In order to pass a test, it is required to obtain at least 56% of correct answers^22^. The examination contains questions regarding:

1. internal diseases, including cardiovascular diseases—39 questions,
2. pediatrics, including neonatology—29 questions,
3. surgery, including trauma surgery—27 questions,
4. obstetrics and gynecology—26 questions,
5. psychiatry—14 questions,
6. family medicine—20 questions,
7. emergency and intensive care medicine—20 questions,
8. bioethics and medical law—10 questions,
9. medical jurisprudence—7 questions,
10. public health—8 questions.

As both models were trained on the data until September 2021 it was decided to evaluate their performance on 3 editions of the Polish Medical Final Examination—Spring 2022 (S22), Autumn 2022 (A22), and Spring 2023 (S23) in two versions—Polish and English. All questions from the previous editions of the examination are available online, along with the average results of medical graduates, detailing overall results, results of graduates who took the exam for the first time, those who graduated in the last 2 years, and those who graduated more than 2 years ago^23^. Besides the content of the question, the correct answers and answer statistics like the index of difficulty (ID) and discrimination power index (DPI) were published. Those indexes were calculated according to the equations presented below^24^:

1 $$ID =(Ns +Ni)/2n$$

2 $$DPI = (Ns-Ni)/n$$

where *n* is the number of examinees in each of the extreme groups (27% of the participants with the best results and 27% with the worst results in the entire test), *Ns*—the number of correct answers to the analyzed task in the group with the best results, *Ni*—the number of correct answers for the analyzed task in the group with the worst results. The index of difficulty takes values from 0 to 1, where 0 means that the task is extremely difficult and 1 means that the task is extremely easy. The discrimination power index assumes values from -1 (for extremely badly discriminating tasks) to 1 (for extremely well discriminating tasks).

For both models, application programming interface (API) provided by OpenAI was used in order to accelerate the process of obtaining answers with *gpt-3.5-turbo* and *gpt-4-0613* models^25^. This allows for providing prompts to GPT models using programming languages and automating the process of obtaining the responses. The analysis was performed with the *temperature* parameter set to 0 and 1 with the *top_p* parameter always set to 1 (default one), as altering both parameters is not recommended^26^. Temperature parameter influences the randomness of the text generated by the model with lower values of this parameter indicate more focused and deterministic responses and higher values make the model's responses more random and creative. Prompts sent through API were the exact questions from the examination without additional comments or context. From each response, the final answer was obtained and saved to the Excel file. If the answer was ambiguous, then the given question was treated as not answered (in other words—incorrectly answered). In the case of GPT-4, questions were taken as an input to the prompt of the models or with API using *gpt-4-0613* model. Final answers from all prompts were stored in Appendices 1 and 2 for GPT-3.5 and GPT-4 respectively.

The accuracy of both models for each test was calculated by dividing the number of correct answers by the number of all questions, which had the correct answer provided. As some questions were invalidated due to inconsistency with the latest knowledge, there were no correct answers for these questions, thus the number of correct answers was divided by the number smaller than 200. Questions which contained image were also excluded. Moreover, the Pearson correlation coefficient between the correctness of the answers, the index of difficulty and the discrimination power index were calculated, and the Mann–Whitney *U* test was conducted to investigate the if there is a difference in those indexes for correct and incorrect answers and Cohen’s d was used to establish effect size (0.2 - small, 0.5 - medium, 0.8 - large)^27^. The overall scores for each examination obtained by LLMs were also compared to the average score obtained by medical graduates who took the exam in the given editions. Consistency of responses depending on the language of the test was also validated by calculating the number of the same answers for each examination. The responses of the models with temperature parameters equal to 0 and 1 were also compared using the Mann–Whitney *U* test for all examination editions and both languages. All questions were asked between the 29th of March and the 14th of August 2023 (ChatGPT March 23 version). The significance level was set at the level of 0.05. For the usage of API, calculations, statistical inference, and visualizations Python 3.9.13 was used. In the process of composing this paper, the authors leveraged Grammarly (Grammarly, Inc.), and GPT-4 to enhance the manuscript's linguistic quality and rectify any grammatical inaccuracies. After employing these tools, the authors reviewed and edited the content as required, thereby accepting complete accountability for the publication's content.

## Results

**Figure 1:**
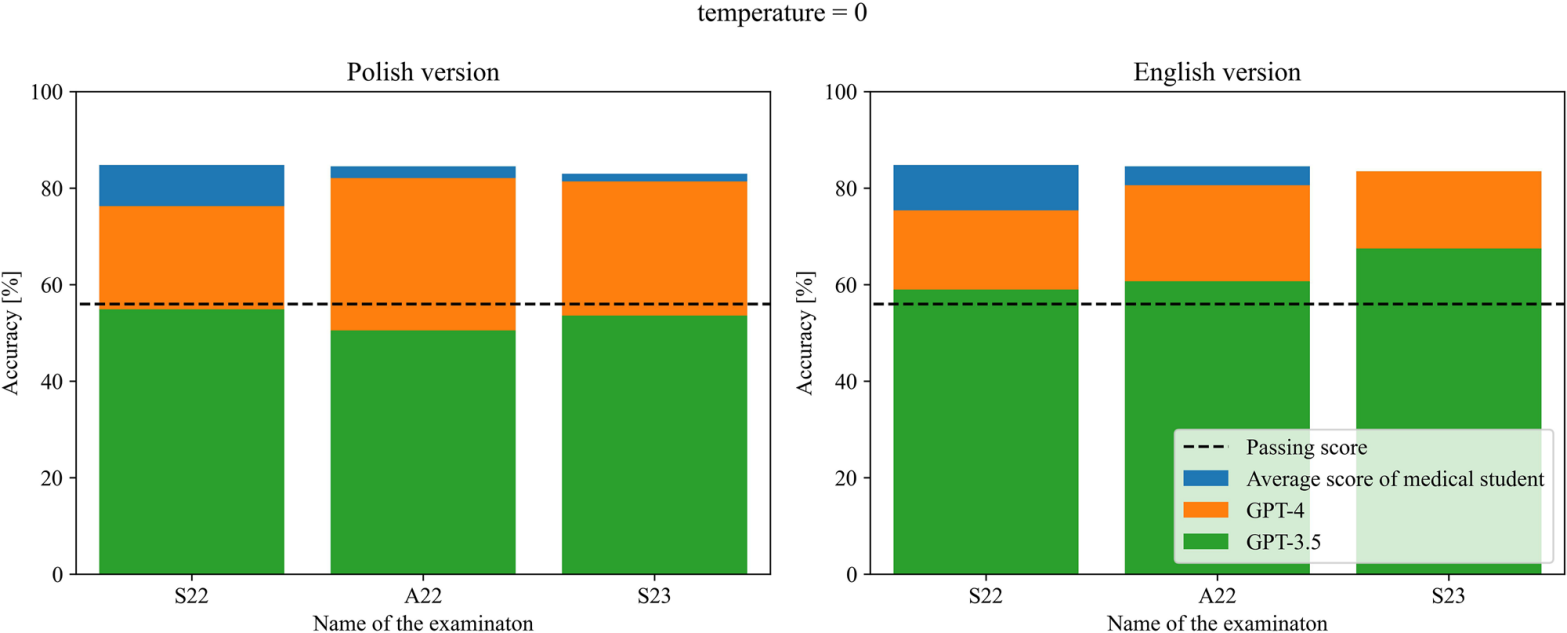
Comparison of the performance of both models along with passing score and average medical graduate score for all three examinations for temperature parameter equal to 0.

**Figure 2:**
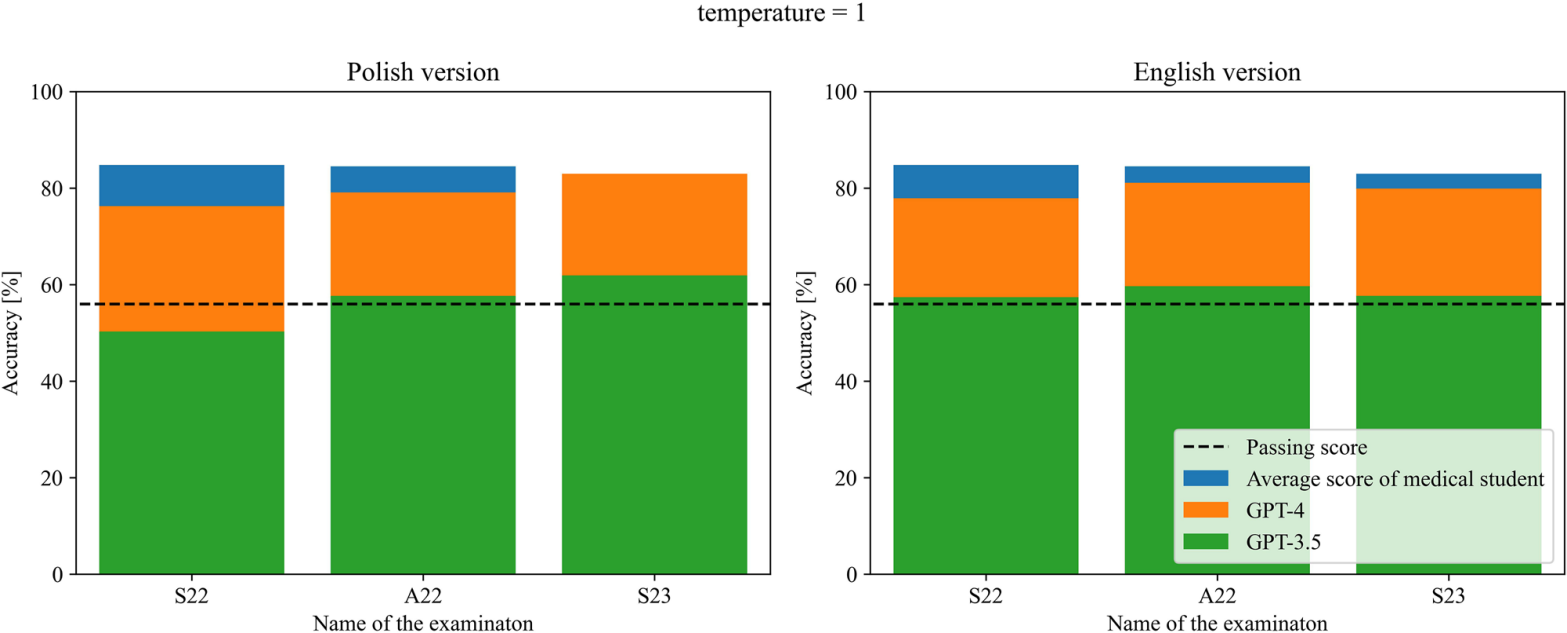
Comparison of the performance of both models along with passing score and average medical graduate score for all three examinations for temperature parameter equal to 1.

GPT-3.5 managed to pass 2 out of 3 versions of examination in Polish in terms of temperature parameter equal to 1, and failed in all versions when this parameter was equal to 0, while passing all versions in English regardless of the temperature parameter. GPT-4 was able to pass all three versions of the examination regardless of language and temperature parameter used. The detailed results obtained by both models are presented in Tables 1 and 2 and visualized in Figs. 1 and 2 for the temperature parameter equal to 0 and 1 respectively.

**Table 1:** Number of correct answers of GPT-3.5 and GPT-4 for each of the undertaken examinations for temperature parameter equal to 0. In the brackets, the number of questions with the given answers and percentage accuracy are provided next to the exam version and the number of correct answers respectively.

Questions language	Model	S22 (195)	A22 (196)	S23 (194)
Polish	GPT-3.5	107 (54.9%)—not passed	99 (50.5%)—not passed	104 (53.6%)—not passed
	GPT-4	148 (76.3%)—passed	161 (82.1%)—passed	158 (81.4%)—passed
English	GPT-3.5	115 (59.0%)—passed	119 (60.7%)—passed	131 (67.5%)—passed
	GPT-4	147 (75.4%)—passed	158 (80.6%)—passed	162 (83.5%)—passed

**Table 2:** Number of correct answers of GPT-3.5 and GPT-4 for each of the undertaken examinations for temperature parameter equal to 1. In the brackets, the number of questions with the given answers and percentage accuracy are provided next to the exam version and the number of correct answers respectively.

Questions language	Model	S22 (195)	A22 (196)	S23 (194)
Polish	GPT-3.5	98 (50.3%)—not passed	113 (57.7%)—passed	120 (61.9%)—passed
	GPT-4	148 (76.3%)—passed	155 (79.1%)—passed	161 (83.0%)—passed
English	GPT-3.5	112 (57.4%)—passed	117 (59.7%)—passed	112 (57.7%)—passed
	GPT-4	152 (77.9%)—passed	159 (81.1%)—passed	155 (79.9%)—passed

**Figure 3:**
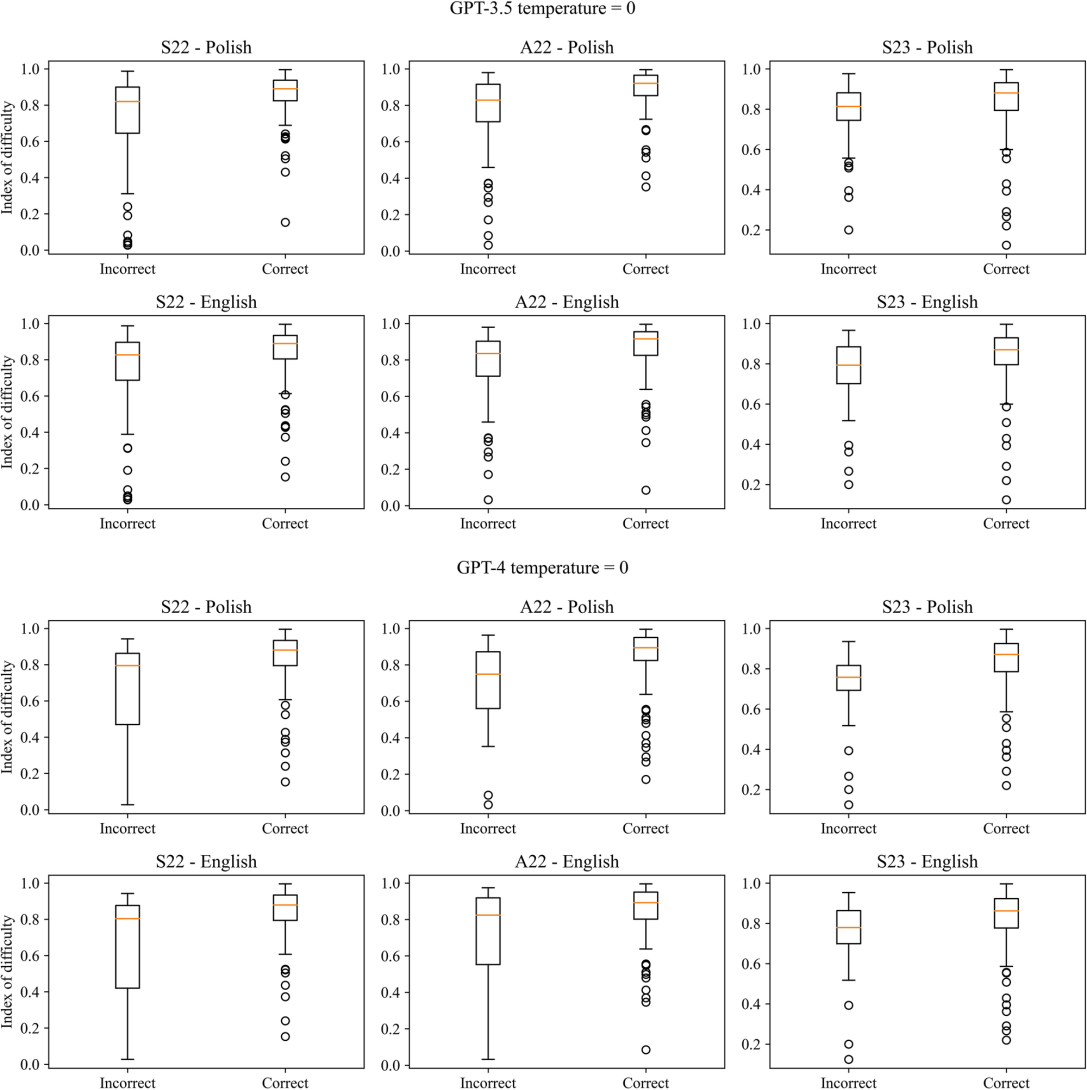
Boxplots of the index of difficulty for the correct and incorrect answers for all three versions of the examination and both languages for temperature parameter equal to 0.

**Figure 4:**
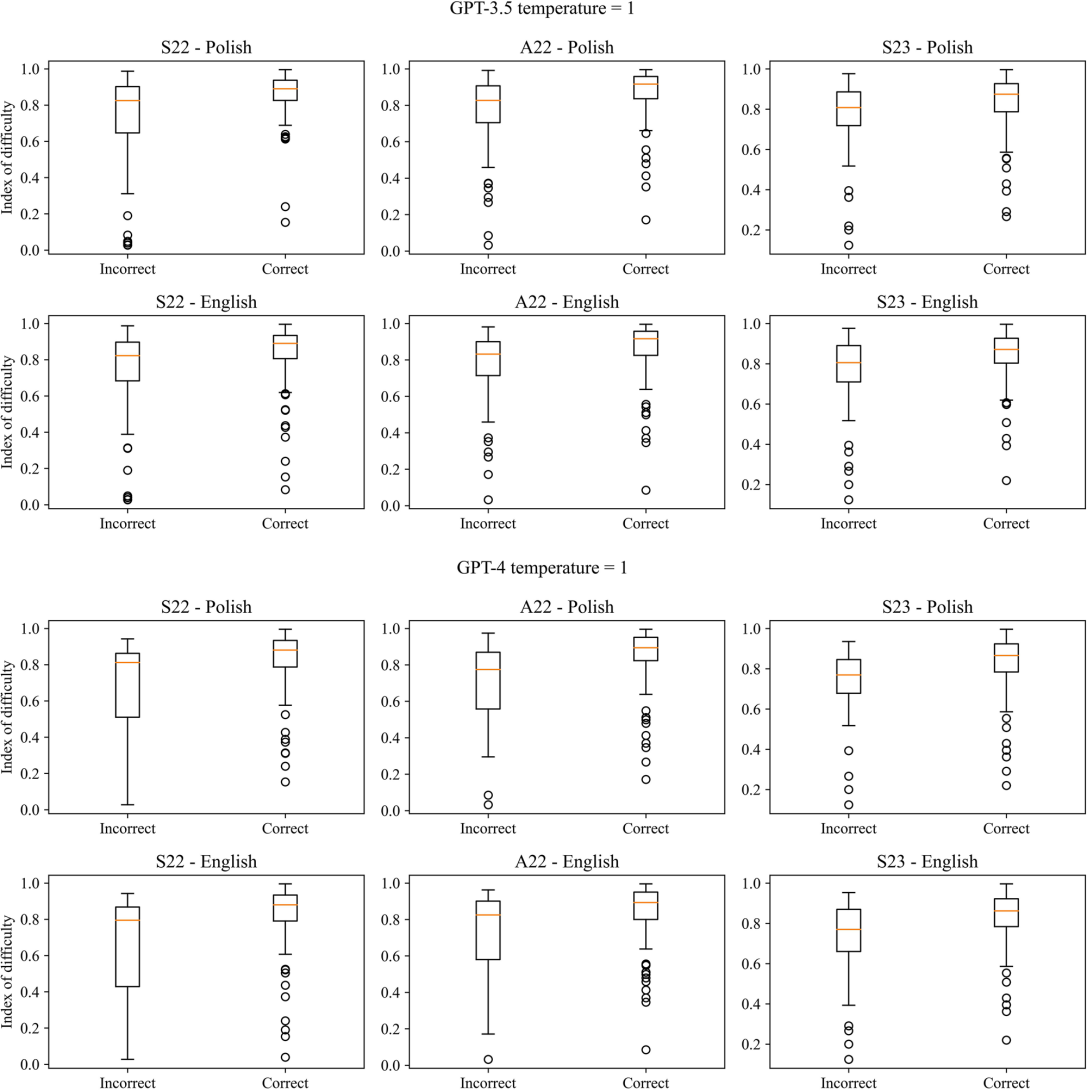
Boxplots of the index of difficulty for the correct and incorrect answers for all three versions of the examination and both languages for temperature parameter equal to 1.

**Figure 5:**
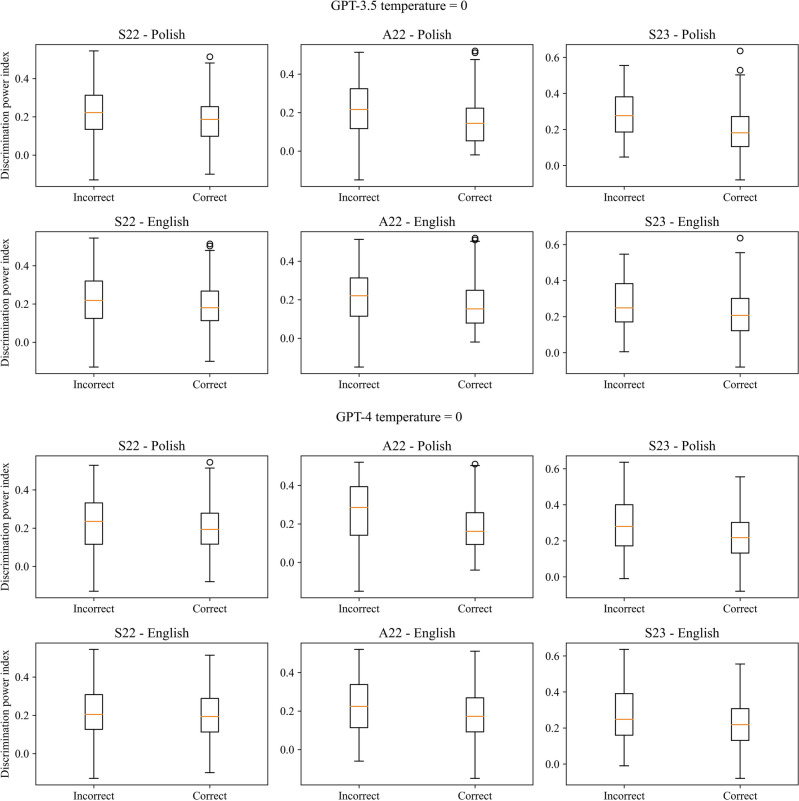
Boxplots of the discrimination power index for the correct and incorrect answers for all three versions of the examination and both languages for temperature parameter equal to 0.

**Figure 6:**
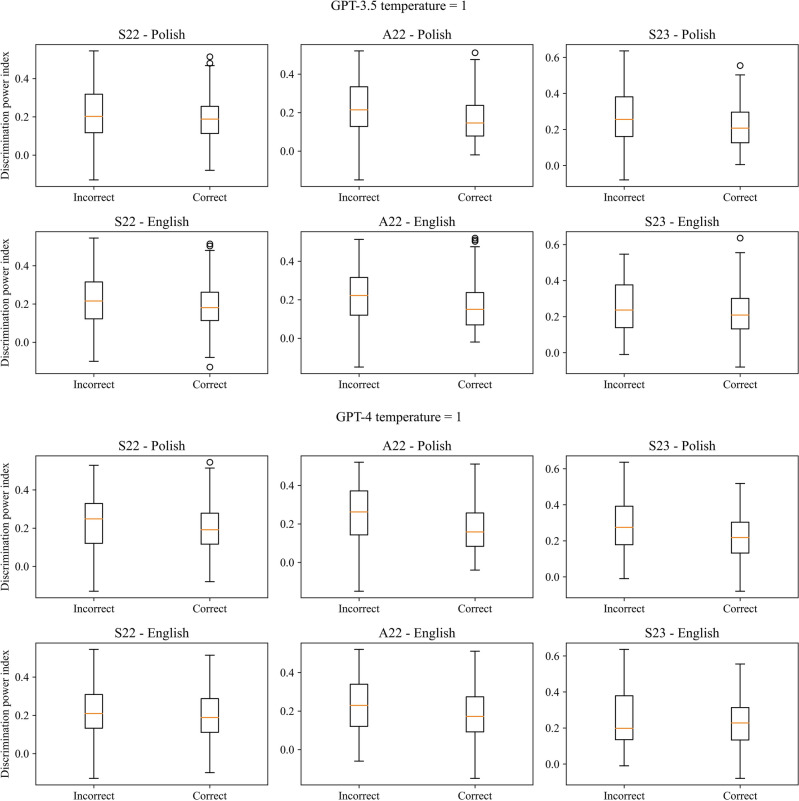
Boxplots of the discrimination power index for the correct and incorrect answers for all three versions of the examination and both languages for temperature parameter equal to 0.

There was a statistically significant positive correlation between the correctness of the answers and the index of difficulty as well statistically significant difference between the index value for correct and incorrect answers in the case of all three exams for both models, temperature parameters and languages, except from Polish version of S23 and GPT-3.5 with temperature set to 0. Cohen’s d for the difference in the index values for correct and incorrect answers indicated a large effect size (> 0.8) in the case of GPT-4, except the English version of A22 for temperature equal to 1, when it was moderate. In the case of GPT-3.5 effect size varies from small to moderate. GPT-4 always obtained a higher value of Cohen’s d compared to GPT-3.5. There was also a statistically significant negative correlation and difference between the correctness of the answers and discrimination power index in the case of the A22 in Polish for both models with both temperature values but only for GPT-3.5 in the case of English version and S23 (only for Polish version) exams for both models. The effect size was small for most cases ranging from 0.026 to 0.690. The results are presented in Tables 3 and 4 for the index of difficulty and Tables 5 and 6 for discrimination power index, for temperature parameters equal to 0 and 1 respectively. The boxplots of the index values depending on the correctness of the answers were visualized in Figs. 3 and 4 for the index of difficulty, and Figs. 5 and 6 for the discrimination power index for temperature parameters equal to 0 and 1 respectively.

**Table 3:** Results of the correlation analysis with Pearson correlation coefficient and obtained p-value given in the brackets along with p-value obtained from the Mann–Whitney *U* test comparing the values of the index of difficulty for correct and incorrect answers for temperature parameter equal to 0.

	S22	A22	S23
Polish			
GPT-3.5			
Pearson correlation coefficient (p-value)	0.333 (< 0.001***)	0.329 (< 0.001***)	0.111 (0.122 ns)
p-value from Mann–Whitney *U* test	< 0.001***	< 0.001***	< 0.001***
Cohen’s d	0.706	0.694	0.224
GPT-4			
Pearson correlation coefficient (p-value)	0.373 (< 0.001***)	0.325 (< 0.001***)	0.311 (< 0.001***)
p-value from Mann–Whitney *U* test	< 0.001***	< 0.001***	< 0.001***
Cohen’s d	0.935	0.886	0.837
English			
GPT-3.5			
Pearson correlation coefficient (p-value)	0.237 (< 0.001***)	0.245 (< 0.001***)	0.198 (0.006**)
p-value from Mann–Whitney *U* test	< 0.001***	< 0.001***	0.001**
Cohen’s d	0.494	0.514	0.430
GPT-4			
Pearson correlation coefficient (p-value)	0.405 (< 0.001***)	0.286 (< 0.001***)	0.224 (0.002**)
p-value from Mann–Whitney U test	< 0.001***	0.002**	0.002**
Cohen’s d	1.022	0.745	0.615

**Table 4:** Results of the correlation analysis with Pearson correlation coefficient and obtained p-value given in the brackets along with p-value obtained from the Mann–Whitney *U* test comparing the values of the index of difficulty for correct and incorrect answers for temperature parameter equal to 1.

	S22	A22	S23
Polish			
GPT-3.5			
Pearson correlation coefficient (p-value)	0.319 (< 0.001***)	0.301 (< 0.001***)	0.174 (0.015**)
p-value from Mann–Whitney *U* test	< 0.001***	< 0.001***	0.004**
Cohen’s d	0.671	0.634	0.362
GPT-4			
Pearson correlation coefficient (p-value)	0.338 (< 0.001***)	0.334 (< 0.001***)	0.299 (< 0.001***)
p-value from Mann–Whitney *U* test	< 0.001***	< 0.001***	< 0.001***
Cohen’s d	0.834	0.861	0.829
English			
GPT-3.5			
Pearson correlation coefficient (p-value)	0.229 (0.001**)	0.245 (< 0.001***)	0.219 (0.002**)
p-value from Mann–Whitney *U* test	< 0.001***	< 0.001***	0.003**
Cohen’s d	0.474	0.511	0.453
GPT-4			
Pearson correlation coefficient (p-value)	0.363 (< 0.001***)	0.273 (< 0.001***)	0.307 (< 0.001***)
p-value from Mann–Whitney *U* test	< 0.001***	0.001**	< 0.001***
Cohen’s d	0.933	0.714	0.802

**Table 5:** Results of the correlation analysis with Pearson correlation coefficient and obtained p-value given in the brackets along with p-value obtained from the Mann–Whitney *U* test comparing the values of the discrimination power index for correct and incorrect answers for temperature parameter equal to 0.

	S22	A22	S23
Polish			
GPT-3.5			
Pearson correlation coefficient (p-value)	− 0.124 (0.083 ns)	− 0.243 (< 0.001***)	− 0.327 (< 0.001***)
p-value from Mann–Whitney *U* test	0.053 ns	< 0.001***	< 0.001***
Cohen’s d	0.251	0.499	0.690
GPT-4			
Pearson correlation coefficient (p-value)	− 0.029 (0.690 ns)	− 0.249 (< 0.001***)	− 0.185 (0.010**)
p-value from Mann–Whitney *U* test	0.410 ns	0.001**	0.031*
Cohen’s d	0.067	0.661	0.482
English			
GPT-3.5			
Pearson correlation coefficient (p-value)	− 0.103 (0.150 ns)	− 0.176 (0.013*)	− 0.182 (0.011*)
p-value from Mann–Whitney *U* test	0.090 ns	0.005**	0.009**
Cohen’s d	0.210	0.363	0.394
GPT-4			
Pearson correlation coefficient (p-value)	− 0.011 (0.877 ns)	− 0.146 (0.041*)	− 0.140 (0.051 ns)
p-value from Mann–Whitney *U* test	0.704 ns	0.072 ns	0.137 ns
Cohen’s d	0.026	0.368	0.380

**Table 6:** Results of the correlation analysis with Pearson correlation coefficient and obtained p-value given in the brackets along with p-value obtained from the Mann–Whitney *U* test comparing the values of the discrimination power index for correct and incorrect answers for temperature parameter equal to 1.

	S22	A22	S23
Polish			
GPT-3.5			
Pearson correlation coefficient (p-value)	− 0.092 (0.200 ns)	− 0.235 (< 0.001***)	− 0.197 (0.006**)
p-value from Mann–Whitney *U* test	0.156 ns	< 0.001***	0.009**
Cohen’s d	0.184	0.487	0.411
GPT-4			
Pearson correlation coefficient (p-value)	− 0.037 (0.607 ns)	− 0.253 (< 0.001***)	− 0.190 (0.008**)
p-value from Mann–Whitney *U* test	0.294 ns	< 0.001***	0.037*
Cohen’s d	0.086	0.636	0.512
English			
GPT-3.5			
Pearson correlation coefficient (p-value)	− 0.098 (0.173 ns)	− 0.191 (0.007**)	− 0.109 (0.129 ns)
p-value from Mann–Whitney *U* test	0.116 ns	0.002**	0.171
Cohen’s d	0.198	0.395	0.221
GPT-4			
Pearson correlation coefficient (p-value)	− 0.050 (0.485 ns)	− 0.151 (0.034*)	− 0.045 (0.555 ns)
p-value from Mann–Whitney *U* test	0.346 ns	0.055 ns	0.929 ns
Cohen’s d	0.121	0.386	0.106

GPT-4 had a higher number of questions with the same given answer regardless of the language of the examination compared to GPT-3.5 for all three versions of the test. The agreement between answers of the GPT models on the same questions in different languages is presented in Tables 7 and 8 for temperature parameters equal to 0 and 1 respectively. There was no statistically significant difference between the results obtained for the same tests and models but with different temperature parameters. In Table 9 the comparison of the results for different temperature parameter values is presented.

**Table 7:** The number of questions on which models provided the same answer regardless of the test language for temperature parameter equal to 0.

	S22	A22	S23
GPT-3.5	136 (88)	121 (87)	128 (92)
GPT-4	162 (131)	161 (143)	170 (150)

In brackets, the number of correct answers with the same response is presented.

**Table 8:** The number of questions on which models provided the same answer regardless of the test language for temperature parameter equal to 1.

	S22	A22	S23
GPT-3.5	116 (79)	113 (85)	111 (86)
GPT-4	159 (133)	162 (143)	168 (148)

In brackets, the number of correct answers with the same response is presented.

**Table 9:** p-values from Mann–Whitney *U* test comparing the correctness of the results obtained for temperature parameter equal to 0 and 1.

	S22	A22	S23
Polish			
GPT-3.5	0.369 ns	0.161 ns	0.108 ns
GPT-4	1.000 ns	0.462 ns	0.710 ns
English			
GPT-3.5	0.763 ns	0.839 ns	0.052 ns
GPT-4	0.566 ns	0.902 ns	0.389 ns

## Discussion

GPT-4 consistently outperformed GPT-3.5 in terms of the number of correct answers and accuracy across three Polish Medical Final Examinations. It indicates a vast improvement in the scope of medical knowledge represented by the GPT-4 model compared to the previous version. For both versions of the model, there is a statistically significant correlation between the accuracy of the answers given and the index of difficulty. Assuming that this index represents the difficulty of the medical issue raised in the given question, as the index is calculated based on the number of correct responses of the best and worst performing participants, it might indicate a lack of in-depth medical knowledge. Additionally, a statistically significant negative correlation and significant difference assessed with the Mann–Whitney *U* test were found between the correctness of the answers and the discrimination power index for almost all models, languages and temperature parameters in the A22 (except from difference for GPT-4 model and English version) and for all settings in the S23 (only Polish version) exams, which might be a sign of the simplicity of the model’s reasoning or the ability to simplify tasks in terms of the medical questions. In all versions of the test, GPT-4 scored slightly below medical student averages, which was equal to 84.8%, 84.5%, and 83.0% for S22, A22 and S23 respectively, except for S23 with temperature parameter equal to 0, where GPT-4 obtained 83.5%. The latest GPT version outperformed students who graduated over 2 years ago for S23 (mean score 156.65) for both languages for temperature values equal 0 and only Polish for temperature equal 1 and those taking A22 as their first exam (mean score 159.57) in case of Polish version and temperature equal to 0. Students who graduated less than 2 years before the examination consistently outperformed both GPT models in both languages. The consistency of the answers between different language versions of the test was much higher for GPT-4 than for GPT-3.5. On average, the most recent model returned identical answers across test languages in 84.3%/83.6% of instances (temperature equal to 0 and 1 respectively), compared to GPT-3.5’s 65.8%/58.1% consistency. This highlights the improvement of the ability of the GPT-4 model to interpret text and encode the knowledge contained in the dataset on which the model was trained. On average, GPT-3.5 exhibited a 9.4% and 1.6% higher accuracy in answering English questions than Polish ones for temperature parameters equal to 0 and 1 respectively. On the contrary, GPT-4 showed a 1.0% higher and 0.2% lower accuracy in Polish over English for temperature parameters equal to 0 and 1 respectively, which contrasts with the evaluation on the Massive Multitask Language Understanding (MMLU) benchmark, where accuracy in Polish was 3.4% lower than in English^6^. The lack of a statistically significant difference between the model results obtained for the temperature parameter equal to 0 and 1 suggests that in this range the value of a given parameter affects rather the overall creativity of the response, but not the representation of medical knowledge encoded in the model. Moreover, there was a notable difference in the responding style between the models as responses from GPT-4 in the vast majority of cases began with the chosen answer (A–E), while responses from GPT-3.5 often were formed in a sentence e.g., “The correct answer is A”.

Our results on the European-based medical final examination are in line with other studies conducted on different tests and languages from North America and Asia, which indicated the improvement of the leverage of the medical knowledge from the training dataset by GPT LLMs alongside with the development of the consecutive versions. Kung et al. evaluated the performance of GPT-3.5 on the USMLE, where GPT-3.5 outperformed its predecessor (GPT-3) with a score near or passing the threshold of 60% accuracy, which is required to pass the exam^8^. Recently, GPT-4 model was also evaluated on USMLE by Nora et al. The newest version of the GPT model outperformed GPT-3.5 with the improvement of its accuracy by over 30 percentage points^15^. In this study, GPT-4 turned out to be superior compared to its previous version and Flan-PaLM 540B model^16^ in the evaluation on other medical benchmarks like MedQA, PubMedQA and MedMCQA. In the study performed by Gilson et al., GPT-3.5 was confronted with the commonly used AMBOSS medical question database and 120 free questions from the National Board of Medical Examiners (NBME)^14^. GPT-3.5 outperformed IntructGPT and GPT-3 models in terms of accuracy by at least 4.9% and 24%, respectively. As shown by Kasai et al., GPT-4 was also able to pass the Japanese Medical Licensing Examinations again outperforming GPT-3.5^7^. This study also highlighted the relationship between the correctness of the answers given by the LLM and the difficulty of the questions, which was also reported in our results. Study performed by Mihalache et al. presented that GPT-3.5 performed the best in the general medicine questions, while obtaining the worst results in the specialized questions^28^. Bhayana et al. demonstrated that GPT-3.5 exhibited superior performance on questions that required low-level thinking compared to those which require high-level thinking^29^. Moreover, the model struggled with questions involving the description of imaging findings, calculation and classification, and applying concepts. Recently, Google and DeepMind presented their LLM PaLM 2 and its medical domain-specific finetuned MedPaLM 2^30,31^. The performance of GPT-4 and MedPaLM 2 on USMLE, PubMedQA, MedMCQA and MMLU appears to be very similar, where both GPT-4 and MedPaLM 2 were superior to each other in an equal number of tests evaluated. In this comparison, it is worth noticing that GPT-4 is a general-purpose model and was not explicitly finetuned for the medical domain.

There may be several potential reasons for the imperfect performance and providing incorrect answers by the tested models. First of all, both models are general-purpose LLMs that are capable of answering questions from various fields and are not dedicated to medical applications. This problem can be addressed by fine-tuning the models, that is, further training them in terms of medical education. As was shown in other studies, a finetuning of LLMs can further increase the accuracy in terms of answering medical questions^32–34^. Currently, OpenAI does not provide finetuning options for GPT-3.5 and GPT-4, but in the future, when this feature becomes available, it is also planned to explore the capabilities of those models after the finetuning on a medical dataset. In order to further increase the model’s accuracy in terms of medical questions the medical databases should be expanded, and instruction prompt tuning techniques could be applied^16^. Furthermore, it is worth noting, there are no details (in the official documents provided by OpenAI) regarding the difference between two version of models in terms of their architecture, number of parameters, size of the training dataset, training methods, etc. The differences are expressed only in the different performance in various benchmarks.

Still, we believe that the appearance of such powerful tools might have a considerable impact on the shape of the public health and medicine of tomorrow^35^. ChatGPT already offered evidence-based advices to public health questions from addiction, interpersonal violence, mental health, and physical health categories^36^. Accurate and validated LLMs with broad medical knowledge can be beneficial for medical students in terms of self-learning, e.g., by generating tailored learning materials, improving physician–patient communication by simulating conversations and clinical reasoning by providing step-by-step explanations of medical cases^37^. This influence will not be restricted to education, but also it might be useful in terms of taking a medical note from a transcript, summarization of test results, or decision-making support^3,37–41^. Moreover, LLMs could also be useful for the personal assistants’ solutions and provide reasonable recommendations in the field of public health e.g., quitting smoking^36^. The importance of prompt engineering (the way of asking questions) should also be emphasized because it affects the quality of the generated answers^42,43^. Also, a recent study has shown that chatbot responses were preferred over physician responses on a social media forum, which shows that AI may strongly improve the quality of medical assistance provided online^44^. However, it is also important to check the authenticity of the responses generated by the GPT model, as it might “hallucinate”, especially regarding provided references^45–47^. Alongside other researchers, we believe that LLMs although they need to be approached with caution, are not a threat to physicians^43^, but can be a valuable tool and will be used more widely in the near future^3,48,49^. As of now, it is necessary to remember, that still a human should be at the end of the processing chain.

While the results of this study demonstrated the potential utility of AI language models in the medical field, several limitations should be acknowledged. First of all, the study focused solely on the Polish Final Medical Examination, which may limit the generalizability of the findings to other medical examinations or languages. What is more, PFME is an A-E test, which means that in some cases the correct answers could be by chance not as the result of the knowledge possessed by the models. Moreover, although GPT-4 outperformed GPT-3.5, the overall accuracy of both models was still suboptimal and worse than the average for medical students. This emphasizes the need for further improvements in LLMs before they can be reliably deployed in medical settings e.g., for self-learning or decision-making support.

## Conclusion

In conclusion, this study highlights the advances in AI language models' performance on medical examinations, with GPT-4 demonstrating superior performance compared to GPT-3.5 regardless of the language and temperature parameter value used. However, there is still considerable room for improvement in their overall accuracy. Future research should focus on finetuning of those models and exploring their potential applications in various medical fields, such as diagnostic assistance, clinical decision support, and medical education. Further tests of LLMs could also include more open questions with evaluation by physicians without prior knowledge of the origins of the answers (if it was created by LLM or a human being).

### Supplementary Information

Supplementary Information 1.

Supplementary Information 2.

## Data Availability

The data used in this study (final answers from all prompts and correct answers) are available in Appendices 1 and 2 for GPT-3.5 and GPT-4 respectively.

## References

[1] Montejo-Ráez A, Jiménez-Zafra SM (2022). Current approaches and applications in natural language processing. Appl. Sci..

[2] Mars M (2022). From word embeddings to pre-trained language models: A state-of-the-art walkthrough. Appl. Sci..

[3] Lee P, Bubeck S, Petro J (2023). Benefits, limits, and risks of GPT-4 as an AI chatbot for medicine. N. Engl. J. Med..

[4] Schulman, J., Wolski, F., Dhariwal, P., Radford, A. & Klimov, O. Proximal policy optimization algorithms. *CoRR*. http://arxiv.org/abs/1707.06347 (2017).

[5] Hendrycks, D. *et al.* Measuring massive multitask language understanding. *CoRR* http://arxiv.org/abs/2009.03300 (2020).

[6] OpenAI. *GPT-4 Technical Report*. (2023).

[7] Kasai J, Kasai Y, Sakaguchi K, Yamada Y, Radev D (2023). Evaluating GPT-4 and ChatGPT on Japanese Medical Licensing Examinations.

[8] Kung TH (2023). Performance of ChatGPT on USMLE: Potential for AI-assisted medical education using large language models. PLOS Dig. Health.

[9] Gencer A, Aydin S (2023). Can ChatGPT pass the thoracic surgery exam?. Am. J. Med. Sci..

[10] Strong E (2023). Chatbot vs medical student performance on free-response clinical reasoning examinations. JAMA Intern. Med..

[11] Beam K (2023). Performance of a large language model on practice questions for the neonatal board examination. JAMA Pediatr..

[12] Wang, X. *et al.* ChatGPT performs on the Chinese national medical licensing examination. 10.21203/rs.3.rs-2584079/v1 (2023). 10.1007/s10916-023-01961-0 37581690

[13] Fang C (2023). How does ChatGPT4 preform on non-English national medical licensing examination? An evaluation in Chinese language. MedRxiv.

[14] Gilson A (2022). How does ChatGPT perform on the medical licensing exams? The implications of large language models for medical education and knowledge assessment. MedRxiv.

[15] Nori, H., King, N., McKinney, S. M., Carignan, D. & Horvitz, E. *Capabilities of GPT-4 on Medical Challenge Problems*. (2023).

[16] Singhal K (2023). Large language models encode clinical knowledge. Nature.

[17] Rao A (2023). Evaluating ChatGPT as an adjunct for radiologic decision-making. MedRxiv.

[18] Brin D (2023). Comparing ChatGPT and GPT-4 performance in USMLE soft skill assessments. Sci. Rep..

[19] BaoZhi S, Yuhong Z (2003). Medical curricula in China and the USA: A comparative study. Med. Teach..

[20] Schulte KL (2017). Credentialing in interventional therapy in Europe: Comparison of curricula including endovascular therapy of arterial diseases. VASA Eur. J. Vasc. Med..

[21] Zavlin D, Jubbal KT, Noé JG, Gansbacher B (2017). A comparison of medical education in Germany and the United States: From applying to medical school to the beginnings of residency. GMS Germ. Med. Sci..

[22] Information about Polish Medical Final Examination. https://www.cem.edu.pl/lek_info.php. Accessed 23 Oct 2023.

[23] Medical Examination Center Web Page. https://cem.edu.pl/. Accessed 23 Oct 2023.

[24] LEK Statistics Description. https://www.cem.edu.pl/aktualnosci/opis_statystyk.pdf. Accessed 23 Oct 2023.

[25] OpenAI Models Documentation. https://platform.openai.com/docs/models/gpt-3-5. Accessed 23 Oct 2023.

[26] OpenAI API Reference. https://platform.openai.com/docs/api-reference/chat/create. Accessed 08 November 2023.

[27] LeCroy CW, Krysik J (2007). Understanding and Interpreting Effect Size Measures. Soc. Work Res..

[28] Mihalache A, Popovic MM, Muni RH (2023). Performance of an artificial intelligence chatbot in ophthalmic knowledge assessment. JAMA Ophthalmol..

[29] Bhayana R, Krishna S, Bleakney RR (2023). Performance of ChatGPT on a radiology board-style examination: insights into current strengths and limitations. Radiology.

[30] Anil, R. *et al.**PaLM 2 Technical Report*. (2023).

[31] Singhal, K. *et al.**Towards Expert-Level Medical Question Answering with Large Language Models*. (2023). 10.1038/s41591-024-03423-7 PMC11922739 39779926

[32] Han, T. *et al.**MedAlpaca: An Open-Source Collection of Medical Conversational AI Models and Training Data*. (2023).

[33] Kormilitzin A, Vaci N, Liu Q, Nevado-Holgado A (2021). Med7: A transferable clinical natural language processing model for electronic health records. Artif. Intell. Med..

[34] Varshney D, Zafar A, Behera NK, Ekbal A (2023). Knowledge graph assisted end-to-end medical dialog generation. Artif. Intell. Med..

[35] Li R, Kumar A, Chen JH (2023). How chatbots and large language model artificial intelligence systems will reshape modern medicine: Fountain of creativity or Pandora’s box?. JAMA Intern. Med..

[36] Ayers JW (2023). Evaluating artificial intelligence responses to public health questions. JAMA Netw. Open.

[37] Ahn S (2023). The impending impacts of large language models on medical education. Korean J. Med. Educ..

[38] Biswas S (2023). ChatGPT and the future of medical writing. Radiology.

[39] Kraljevic Z (2021). Multi-domain clinical natural language processing with MedCAT: The medical concept annotation toolkit. Artif. Intell. Med..

[40] Rao A (2023). Assessing the utility of ChatGPT throughout the entire clinical workflow. MedRxiv.

[41] Cascella M, Montomoli J, Bellini V, Bignami E (2023). Evaluating the feasibility of ChatGPT in healthcare: An analysis of multiple clinical and research scenarios. J. Med. Syst..

[42] Short CE, Short JC (2023). The artificially intelligent entrepreneur: ChatGPT, prompt engineering, and entrepreneurial rhetoric creation. J. Bus. Ventur. Insights.

[43] Harris E (2023). Large language models answer medical questions accurately, but can’t match clinicians’ knowledge. JAMA.

[44] Ayers JW (2023). Comparing physician and artificial intelligence chatbot responses to patient questions posted to a public social media forum. JAMA Intern. Med..

[45] Sallam M (2023). ChatGPT utility in healthcare education, research, and practice: Systematic review on the promising perspectives and valid concerns. Healthcare.

[46] Alkaissi H, McFarlane SI (2023). Artificial hallucinations in ChatGPT: Implications in scientific writing. Cureus.

[47] Eysenbach G (2023). The role of ChatGPT, generative language models, and artificial intelligence in medical education: A conversation with ChatGPT and a call for papers. JMIR Med. Educ..

[48] Cheng K (2023). Artificial intelligence in sports medicine: Could GPT-4 make human doctors obsolete?. Ann. Biomed. Eng..

[49] Lee H (2023). The rise of ChatGPT: Exploring its potential in medical education. Anat. Sci. Educ..

